# Case Report: Triple-negative breast cancer with brain and meningeal metastases exhibits spatiotemporal heterogeneity in terms of HER2 expression

**DOI:** 10.3389/fonc.2025.1599148

**Published:** 2025-06-19

**Authors:** Xubin Wang, Zhiyun Weng

**Affiliations:** The Affiliated Yueqing Hospital of Wenzhou Medical University, Yueqing, Zhejiang, China

**Keywords:** human epidermal growth factor receptor 2, trastuzumab deruxtecan, case report, meningeal carcinomatosis, triple-negative breast cancers

## Abstract

Meningeal carcinomatosis (MC) is a distinct form of brain metastasis (BM) that occurs in patients with advanced triple-negative breast cancer (TNBC). The occurrence of BM typically indicates a poor prognosis. Spatial heterogeneity in HER2 expression is relatively common in breast cancer cases; however, the emergence of both temporal and spatial heterogeneity within the brain parenchyma and cerebrospinal fluid (CSF) is exceedingly rare. Thus, the phenomenon warrants further investigation. Herein, we report a case of advanced TNBC with BM and MC. HER2 was expressed in the CSF and exhibited spatial and temporal heterogeneity. The CSF was analyzed using immunohistochemistry and flow cytometry, confirming the presence of HER2-positive tumor cells in the patient’s CSF. MC was effectively controlled after treatment with trastuzumab deruxtecan (T-DXd). Relevant literature was reviewed to analyze the reasons for this phenomenon. In this case, the spatial and temporal heterogeneity of the HER2 receptors observed in the CSF suggests that BM may be driven by the synergistic interaction of multiple subclonal tumor cells.

## 1 Introduction

Breast cancer has a very heterogeneous disease presentation. The most common classification is based on four major biomarkers, namely the estrogen receptor (ER), the progesterone receptor (PR), Ki67, and human epidermal growth factor receptor 2 (HER2), which are divided into four subtypes based on their immunohistochemical expression. Breast cancer that does not express ER, PR, and HER2 is defined as triple-negative breast cancer (TNBC) (1). Gene signatures differ greatly between breast cancer subtypes, depending on their signaling pathways, exhibiting differences in metastatic site preference (2). Brain metastases (BMs) are common metastatic sites in advanced breast cancer and are most common in HER2-positive and TNBC subtypes, with an incidence of 20%–30% (3). The median time from diagnosis to BM development for these two subtypes is 28–36 months. Once BM occurs, it often presages an extremely poor prognosis (4). Meningeal carcinomatosis (MC) is a specific site of BM. Approximately 2%–5% of patients with breast cancer develop MC, which can result in a severely poor prognosis (5). Currently, there are four common treatments for BM: intrathecal chemotherapy, radiotherapy, surgery, and systemic treatment with anticancer drugs (6).

Herein, we report a case of advanced TNBC with BM and MC. HER2 expression was not detected in the primary mammary tumor or brain parenchyma but was identified in cerebrospinal fluid (CSF) tumor cells, demonstrating spatial and temporal heterogeneity. MC was effectively controlled after treatment with trastuzumab deruxtecan (T-DXd).

## 2 Case presentation

### 2.1 Chief complaints

A 42-year-old woman presented to the emergency department with meningeal metastases from breast cancer, headache, vomiting, and paroxysmal confusion.

### 2.2 History of the present illness

In January 2024, the patient developed a gradually aggravated headache, and a lumbar puncture was performed at another hospital. At that time, the CSF pressure increased to 260 mmH_2_O, indicating meningeal metastasis of the breast cancer. The patient was dehydrated to reduce the intracranial pressure, and the treatment effect was poor. The disease progressed rapidly and gradually resulted in decreased muscle strength in the extremities up to grade IV.

### 2.3 History of past illness

In November 2021, the patient underwent right mastectomy and lymph node dissection of a breast mass found during a physical examination. Postoperative pathology revealed TNBC; the histological type was invasive ductal carcinoma, the tumor size was 5 cm × 4 cm × 4 cm with four axillary lymph node metastases, the resection margin of the tumor was negative, and a cancer thrombus was visible in the vessel. As the patient’s tumor stage reached pT3N2M0, she received four courses of epirubicin combined with cyclophosphamide, followed by four courses of docetaxel (AC-T). After the completion of chemotherapy, local radiotherapy was continued, and S-1 was used as a consolidation regimen for 6 months. During follow-up in April 2023, the patient was found to have BM in both the left and right frontal lobes. The left lesion was surgically resected, and the right metastatic lesion was treated with stereoscopic radiotherapy. Immunohistochemistry was used to detect the expression of HER2 in the resected brain tissue, and the result was considered zero. The patient received seven courses of utidelone (UTD1) from May 2023 to October 2023. In November 2023 and January 2024, the patient underwent CyberKnife therapy due to the recurrence of a new lesion in the left frontal lobe. During the two periods of CyberKnife treatment, the patient received one course of chemotherapy with gemcitabine, cisplatin, and pembrolizumab. In January 2024, the patient presented with clinical symptoms of meningeal metastasis. As her condition continued to deteriorate, she was referred to our hospital.

### 2.4 Personal and family history

The patient received long-term entecavir tablets for chronic viral hepatitis B infection and had no family history of tumor-related diseases.

### 2.5 Physical examination

The patient presented with paroxysmal confusion and weakness of the extremities, with normal muscle tone.

### 2.6 Laboratory examinations

**Figure 1:**
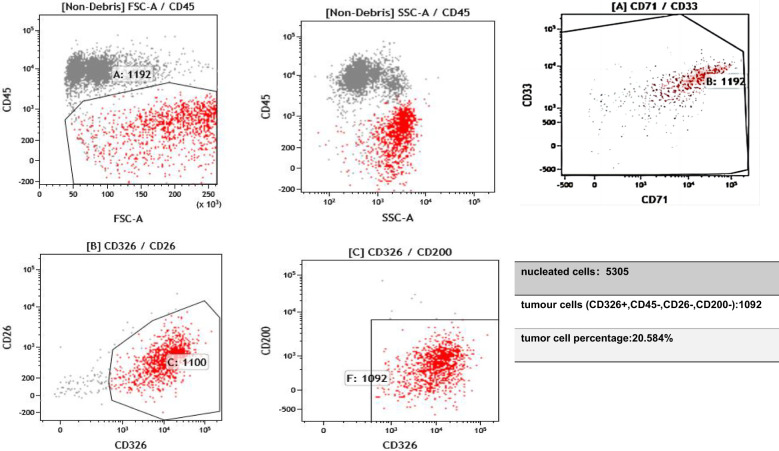
On 11 February 2024, flow cytometry analysis of the cerebrospinal fluid identified a population of epithelial cells positive for CD326 and negative for CD45. CD45 is expressed in hematopoietic cells but is absent in tumor cells. CD33 and CD71 serve to exclude myeloid cells and nucleated red blood cells. Epithelial-derived tumor cells typically express CD326 (EpCAM). CD26 and CD200 are expressed on mesothelial cells and can help distinguish mesothelial cells from tumor cells.

In February 2024, the patient’s CSF was tested after the symptoms of MC developed; the results showed proliferating and infiltrating atypical cells, and 6% of the cells were HER2(+++). Flow cytometry testing of the CSF, using the epithelial cell-specific marker CD326 and the leukocyte marker CD45, suggested that the proportion of tumor cells in the CSF was 20% (**Figure 1**). The proportion decreased to zero after treatment with T-DXd.

### 2.7 Pathological diagnosis

Histopathology of the right breast revealed invasive ductal carcinoma, histological grade III, with tumor size 5 cm × 4 cm × 4 cm. Immunohistochemical results were as follows: CD31 (vascular endothelial +), CK5/6 (neoplastic cells +), calponin (−), D2-40 (lymphatic +), E-cd (+), ER (−), HER2(0), Ki67 (70%), P63 (−), and PR (−).

### 2.8 Imaging examinations

**Figure 2:**
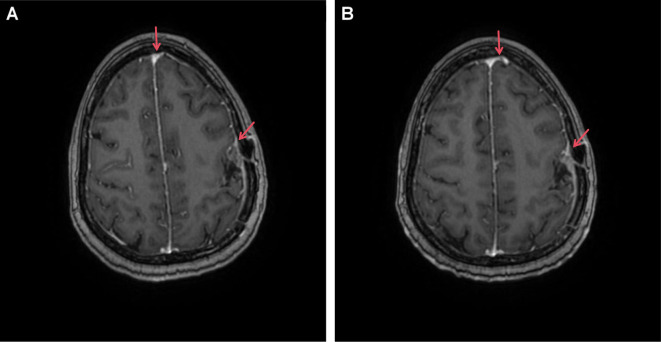
Enhanced MRI image of our patient with brain metastasis undergoing T-DXd treatment. **(A)** T1 image (20 February 2024); **(B)** T1 image (14 April 2024). Red arrows highlight areas of tumor presence along with meningeal thickening and enhancement.

Magnetic resonance imaging revealed postoperative changes in the skull, patchy heterogeneous enhancement of the left frontoparietal junction, and meningeal thickening (**Figure 2**).

### 2.9 Final diagnosis

Based on the available information, the patient was diagnosed with a triple-negative breast malignancy (pT3N2M0). The stage after recurrence was rTxNxM1 (brain and meningeal metastases).

### 2.10 Treatment

The patient declined whole-brain radiation and, after ruling out any contraindications, intrathecal thiotepa was administered twice weekly for disease control. Based on the findings from the DESTINY-Breast04 trial, which demonstrated the clinical benefit of T-DXd in patients with low HER2 expression, we adjusted the treatment strategy for this case. Following detailed discussion with the patient and confirmation of HER2 expression (6% of tumor cells were HER2+++, as per immunohistochemistry of the CSF), the patient consented to off-label therapy with T-DXd at a dose of 300 mg every 3 weeks that was initiated in February 2024.

### 2.11 Outcome and follow-up

**Figure 3:**
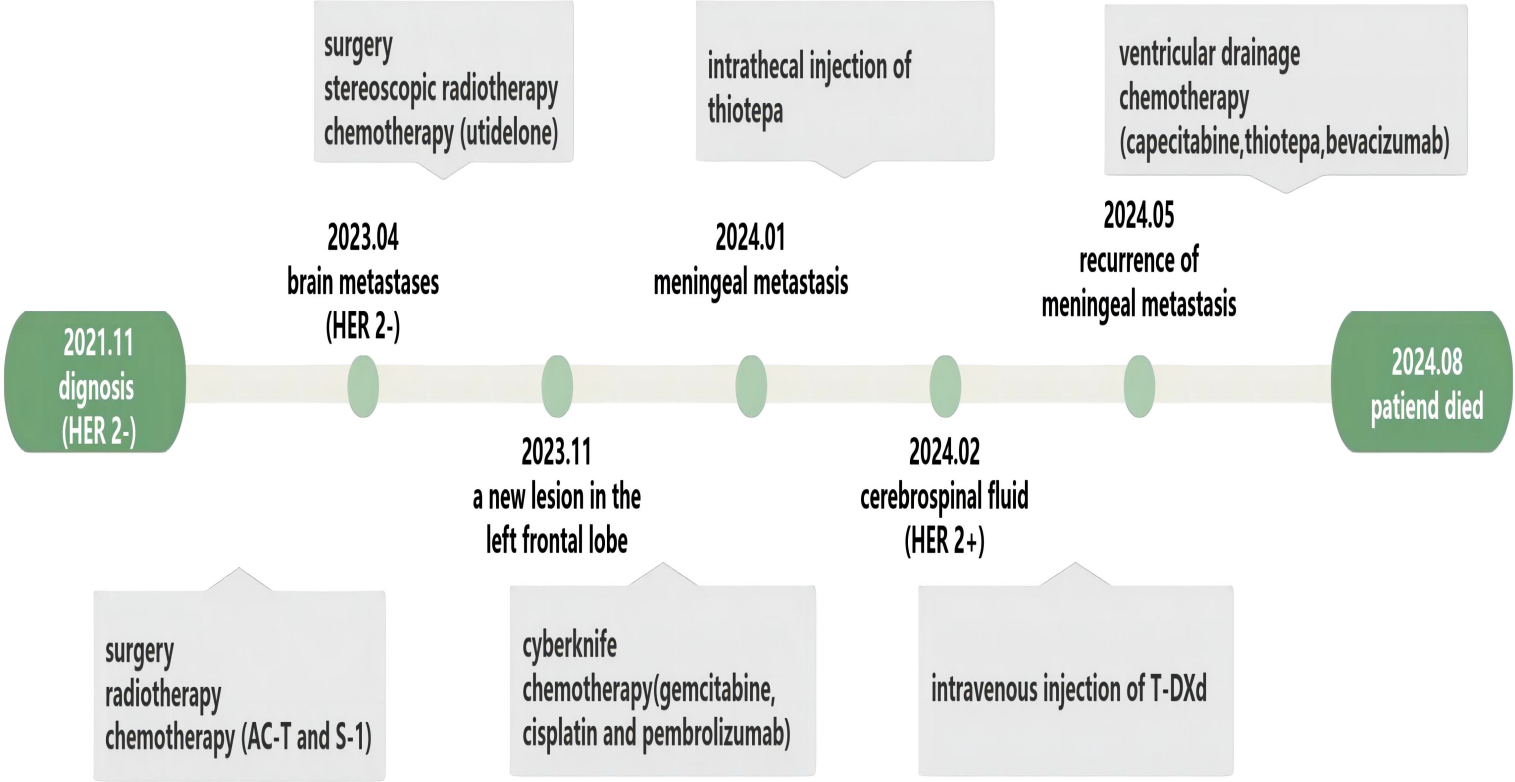
The treatment course of our patient.

After one course of T-DXd, the patient’s headache significantly improved, autonomous consciousness was recovered, and limb muscle strength returned to normal. After three courses of T-DXd, we reviewed the flow cytometry examination of the CSF, and the results showed that the proportion of tumor cells had decreased to 0. For a period of 3 months, the patient reported good quality of life and normal daily functioning. However, this state was not sustained, and the patient’s condition rapidly deteriorated 1 month later. Thiotepa was then administered, but it only delayed disease progression. In August 2024, after 3 months of palliative treatment, the patient chose to refuse further treatment due to aggravation of the disease (**Figure 3**).

## 3 Discussion

MC is a devastating metastatic site of cancer that is common in advanced breast cancer, and with the improvements in patient survival rate, this proportion will gradually increase (7, 8). The mean overall survival of patients with MC is 2–4 months, with rapid deterioration occurring within 4–6 weeks in untreated patients (9). TNBC is associated with an elevated risk of MC, and its prognosis is worse than that of other types of breast cancer (7). Given the poor MC prognosis, enabling early diagnosis could optimize the timing of therapeutic interventions for patients. Current clinical diagnosis of MC primarily relies on MRI and CSF cytology, yet these methods often fail to detect the disease at early stages (10–12). For patients with high-risk MC, advanced liquid biopsy technologies, such as CellSearch^®^ technology, modified fast aneuploidy screening test-sequencing system (mFAST-SeqS), and ultra-low-pass whole-genome sequencing (ulpWGS), applied to CSF analysis may improve diagnostic sensitivity and ultimately enhance patient survival outcomes (13–15). Currently, systemic therapy remains critically important in the management of breast cancer with BM. Notably, investigative chemotherapy, such as antibody–drug conjugates (ADCs) and tyrosine kinase inhibitors (TKIs), has demonstrated particularly encouraging efficacy in improving survival outcomes for these patients (**Table 1**). Our patient exhibited a unique pathological characteristic. The patient’s primary tumor site and BM site were HER2(0); however, when the patient had MC, 6% of the cells in the CSF were positive for HER2 (+++). This rare phenomenon reflects the spatiotemporal heterogeneity of tumors.

**Table 1:** Upcoming clinical trials for breast cancer with BM.

Compound	Trial	Phase and status	Parameters	Comment
T-DXd	DESTINY-Breast12 NCTO4739761	Complete	HER2+ advanced or metastatic breast cancer. Received ≤2 prior lines of therapy in the metastatic setting	Patients with BM Median PFS 17.3 months
Trastuzumab emtansine + tucatinib	HER2CLIMB-02 NCT02614794	Complete	HER2+ advanced or metastatic BC with progression after trastuzumab and taxane in any setting	Patients with BM Median PFS 7.8 months
Trastuzumab emtansine + neratinib	TBCRC-022 NCTO1494662	Complete	HER2+ breast cancer with BM	Group 4A Median PFS 5.3 months Group 4B Median PFS 4.1 months Group 4C Median PFS 4.1 months
Datopotamab deruxtecan	TUXEDO-2	Phase II, currently recruiting	TNBC with BM	No study results posted
Utidelone + bevacizumab	U-BOMB NCT05357417	Complete	HER2− breast cancer with BM	Median PFS 7.7 months
Pyrotinib + capecitabine	PERMEATE NCT03691051	Complete	HER2+ breast cancer with BM	Median PFS 11.3 months

To date, knowledge of the mechanism of BM from breast cancer is still limited, but the process can be roughly summarized as follows: gene expression activates certain signaling pathways that allow tumor extravasation after the dynamic remodeling of the extracellular matrix (16); tumor cells infiltrate into peripheral blood vessels; the interaction between the microenvironment of the blood–brain barrier (BBB) and metastatic breast cancer cells establishes a metastatic niche that promotes BM; and cells with a particular genetic profile colonize the new soil of the brain (17). MC, as a distinct subtype of BM, exhibits a lower incidence rate and demonstrates highly diverse metastatic routes. However, different cancer types may exhibit a predilection for specific pathways (18). The pathways of cancer cell metastasis in MC include the following: 1) cancer cells invade arachnoid granulations through venous sinuses; 2) tumors within the brain parenchyma breach the glia limitans to enter perivascular (Virchow–Robin) spaces; 3) tumor cells in the dura mater invade via meningeal lymphatic vessels; 4) cancer cells in arterial circulation cross through fenestrated vessels and choroid plexus epithelial tight junctions; 5) cancer cells migrate along nerve bundles to reach the leptomeninges; 6) cancer cells invade via retrograde flow through Batson’s valveless venous plexus; and 7) cancer cells from bone metastatic lesions infiltrate through bridging veins. Interestingly, the last pathway has been demonstrated and widely discussed in breast cancer (19). The leptomeningeal environment is nutrient-deprived and hypoxic. Upon metastasizing to the leptomeninges, cancer cells must adapt to these harsh conditions. Examples include remodeling the local microenvironment by upregulating complement component C3 (C3) or lipocalin-2 (LCN2) to promote survival (20, 21). Metabolic adaptation is also critical, such as leveraging glycolysis under hypoxia and oxidative phosphorylation (12, 22). Discordance in HER2 receptor expression exists between primary breast cancer and BM. Some tissues show subtype switching, and the proportion is up to one-third in BM (23, 24). This phenomenon is not difficult to understand when considering BM formation as a process of tumor cell selection and evolution. At the same time, attention must be paid to tumor heterogeneity.

Spatial heterogeneity refers to phenotypic and genomic differences in different regions of a tumor, whereas temporal heterogeneity refers to the emergence of new biological characteristics of metastatic lesions after tumor progression (25). The former reflects the coexistence of multiple subclones of tumor cells (26, 27), while the latter is often more complex and usually related to the pattern of tumor evolution and the seeding pattern of metastasis. The most prevalent form of spatial heterogeneity observed in breast cancer is the expression of HER2 receptors, accounting for 23% of cases (28). Intratumor HER2 heterogeneity has been linked to a shorter duration of disease-free survival, particularly in patients with TNBC (29, 30). Temporal heterogeneity between primary and metastatic tumors can be explained by two models of tumor cell evolution. These two evolutionary models exhibit linear and parallel progressions (31). The linear progression of metastatic tumors is driven by dominant clones selectively derived from the primary tumor in response to therapeutic pressure. Genetic disparities between primary and metastatic tumors are minimal (32). A parallel progression model often occurs in cases where the primary tumor has multiple subclones that disseminate early during treatment and undergo a quiescent phase before undergoing separate clonal evolution, resulting in significant genetic divergence between primary and metastatic tumors (33).

Before the implementation of single-cell and multiregion sequencing techniques, it was widely acknowledged that metastasis was predominantly driven by a single subclone (34). However, technological advances have led to new theories, including polyclonal seeding and metastatic cross-seeding, in which clusters of tumor cells composed of multiple subclones may be more effective than monoclonal cells in the formation of metastatic tumors (34).

A key limitation of this study was the inability to perform single-cell sequencing, thereby restricting our capacity to fully characterize the observed tumor heterogeneity. However, when HER2 receptor heterogeneity is present in the brain tissue and CSF, it is reasonable to believe that BM arises from the synergistic interaction of multiple subclonal tumor cells.

## 4 Conclusion

With the increasing adoption of rapid and affordable sequencing technology and the enrichment of tumor treatment methods, we will gain a deeper understanding of the heterogeneity of metastatic tumors. The development of personalized treatment strategies for each patient will most likely become the norm in the future.

## Data Availability

The original contributions presented in the study are included in the article/supplementary material. Further inquiries can be directed to the corresponding author.
